# *SPTBN2*, a New Biomarker of Lung Adenocarcinoma

**DOI:** 10.3389/fonc.2021.754290

**Published:** 2021-10-20

**Authors:** Chunli Wu, Bo Dong, Lan Huang, Yafei Liu, Guanchao Ye, Shihao Li, Yu Qi

**Affiliations:** ^1^ Department of Thoracic Surgery, The First Affiliated Hospital of Zhengzhou University, Henan, China; ^2^ Biotherapy Center, The First Affiliated Hospital of Zhengzhou University, Henan, China

**Keywords:** lung adenocarcinoma, bioinformatics analysis, SPTBN2, migration, proliferation, invasion

## Abstract

**Objectives:**

The roles played by β-III-spectrin, also known as spectrin beta, non-erythrocytic 2 (*SPTBN2*), in the occurrence and development of lung adenocarcinoma (LUAD) have not been previously examined. Our study aimed to reveal the relationship between the *SPTBN2* expression and LUAD.

**Materials and Methods:**

Twenty pairs of LUAD tissues and adjacent tissues were collected from patients diagnosed and treated at the Thoracic Surgery Department of The First Affiliated Hospital of Zhengzhou University from July 2019 to September 2020. RNA sequencing (RNA-seq) analysis determined that the expression of *SPTBN2* was higher in LUAD samples than in adjacent normal tissues. The expression levels of *SPTBN2* were examined in various databases, including the Cancer Cell Line Encyclopedia (CCLE), Gene Expression Omnibus (GEO), and Human Protein Atlas (HPA). The Search Tool for the Retrieval of Interacting Genes (STRING) online website was used to examine protein–protein interactions involving *SPTBN2*, and the results were visualized by Cytoscape software. The Molecular Complex Detection (MCODE) plug-in for Cytoscape software was used to identify functional modules of the obtained protein–protein interaction (PPI) network. Gene enrichment analysis was performed, and survival analysis was conducted using the Kaplan–Meier plotter. The online prediction website TargetScan was used to predict SPTBN2-targeted miRNA sequences by searching for *SPTBN2* sequences. Finally, we verified the expression of *SPTBN2* in the obtained tissue samples using real-time fluorescence quantitative polymerase chain reaction (RT-qPCR). The human lung cancer cell lines A549 and H1299 were selected for the transfection of small interfering RNA (siRNA) targeting *SPTBN2* (si-SPTBN2), and the knockdown efficiency was evaluated by RT-qPCR. The cellular proliferation, migration, and invasion capacities of A549 and H1299 cells were determined using the cell counting kit-8 (CCK-8) proliferation assay; the wound-healing assay and the Transwell migration assay; and the Matrigel invasion assay, respectively.

**Results:**

The expression of *SPTBN2* in non–small cell lung cancer (NSCLC) ranked 13th among cancer cell lines based on the CCLE database. At the mRNA and protein levels, the expression levels of *SPTBN2* were higher in LUAD tissues than in normal lung tissues. Kyoto Encyclopedia of Genes and Genomes (KEGG) pathway analysis revealed that proteins related to *SPTBN2* were enriched in apoptotic and phagosomal pathways. Kaplan–Meier survival analysis revealed that *SPTBN2* expression was significantly related to the prognosis of patients with LUAD. The TargetScan database verified that miR-16 was a negative regulator of *SPTBN2* mRNA expression. The results of the CCK-8 cell proliferation assay revealed that *SPTBN2* knockdown significantly inhibited the cell proliferation abilities of A549 and H1299 cells. The wound-healing assay indicated that *SPTBN2* knockdown resulted in reduced migration after 48 h compared with the control group. The Transwell migration and invasion test revealed that the migration and invasion abilities were greatly decreased by *SPTBN2* knockdown compared with control conditions.

**Conclusion:**

We uncovered a novel gene, *SPTBN2*, that was significantly upregulated in LUAD tissues relative to normal tissue expression. *SPTBN2* is highly expressed in LUAD, positively correlated with poor prognosis, and can promote the proliferation, migration, and invasion of LUAD cells.

## 1 Introduction

Lung cancer is currently the most commonly diagnosed cancer, accounting for 11.6% of all diagnosed cancer cases, and represents the leading cause of cancer-related mortality (18.4% of overall cancer mortality) worldwide (1). *SPTBN2* encodes β-III-spectrin, also known as spectrin beta, non-erythrocytic 2, which is expressed throughout the cell body, particularly in the dendritic tree of Purkinje cells (2). The biological function of *SPTBN2* was first reported in association with spinocerebellar ataxia (3). In a recent study, the occurrence of various tumor types has been associated with *SPTBN2* (4). However, the relationship between *SPTBN2* and lung adenocarcinoma (LUAD) has never been reported. We conducted this study to explore the relationship between *SPTBN2* expression and LUAD and examine the roles played by *SPTBN2* in the proliferation, migration, and invasion of LUAD cells.

## 2 Materials and Methods

### 2.1 Samples and RNA Sequencing

We obtained 20 pairs of LUAD samples and tumor-adjacent normal tissues from our hospital. The criteria for patient inclusion were as follows. 1) The patient has not been treated for LUAD at other hospitals. 2) The patient was diagnosed with LUAD and underwent radical surgery. 3) The postoperative specimens were confirmed to be LUAD by immunohistochemistry performed by the Pathology Department. 4) The patient provided informed consent. Obtained specimens were immediately marked with the patient’s name and separated into centrifuge tubes. All tissues were stored at −80°C until use. We performed RNA-seq to determine that *SPTBN2* expression levels in LUAD samples compared with those in tumor-adjacent normal tissues. This study was approved by the Ethics Committee of the First Affiliated Hospital of Zhengzhou University (No. 2019-KY-255).

### 2.2 Bioinformatics Analysis

#### 2.2.1 Cancer Cell Line Encyclopedia Analysis

The Cancer Cell Line Encyclopedia (CCLE) database contains the RNA-seq data from 1,457 cancer cell lines, maintained by the Broad Institute of MIT and Harvard (5). We analyzed mRNA expression levels of *SPTBN2* across various cancer cell lines.

#### 2.2.2 Gene Expression Omnibus Database Analysis

The Gene Expression Omnibus (GEO) database includes written descriptions of experimental designs, sample attributes, and methodologies for studies that performed high-throughput gene expression and genomics analyses (6). We used the GEO database to compare the mRNA expression levels of *SPTBN2* in tumor tissues with those in normal lung tissues: GSE10072 (58 LUAD tissues and 49 normal tissues) (7) and GSE32863 (58 LUAD tissues and 58 normal tissues) (8). Differences in *SPTBN2* expression were then verified using the GSE75037 (83 LUAD tissues and 83 normal tissues) (9) and GSE7670 (28 LUAD tissues and 30 normal tissues) (10) datasets. Each dataset was preprocessed using the original authors’ approach. The Gene Expression Profiling Interactive Analysis (GEPIA) database was used to perform gene expression analysis using tumor samples and normal samples obtained from The Cancer Genome Atlas (TCGA) and Genotype-Tissue Expression (GTEx) databases (11). The mRNA expression levels in LUAD and normal samples were verified by the GEPIA database.

#### 2.2.3 Protein–Protein Interaction Network and Gene Set Enrichment Analysis

Data for *SPTBN2* protein–protein interactions (PPIs) were obtained using the Search Tool for the Retrieval of Interacting Genes (STRING) online website and visualized by Cytoscape software. Functional modules in the PPI network were analyzed using the Molecular Complex Detection (MCODE) plug-in in Cytoscape software, with the following settings: degree cutoff = 2; node score cutoff = 0.2; K-score = 2; and max depth = 100. Genes with a high degree of connectivity within the functional modules were regarded as core genes. The top 100 genes identified as strongly correlated with *SPTBN2* in the TCGA database were enriched and analyzed.

#### 2.2.4 The Human Protein Atlas Analysis

This study used immunohistochemistry (IHC) staining data to analyze *β-III spectrin* expression levels between LUAD tissues and normal tissues from the Human Protein Atlas (HPA) database (12–14). Furthermore, we analyzed the correlation between *SPTBN2* mRNA levels and patient prognosis. The expression levels were shown as four sorts: not detected, low, medium, and high. The proportion of stained cells (<25%, 25%–75%, and >75%) and the intensity of staining (negative, weak, moderate, and strong) made up the scoring system.

#### 2.2.5 KM-PLOTTER Survival Analysis Online

The TCGA database contains information for various human cancer types, including clinical data, genome variations, mRNA expression, miRNA expression, methylation, and other data, which can be used to study survival and analyze differences between patients diagnosed with various tumors and healthy people in addition to performing correlation analyses between omics data and clinical data (15). Samples with *SPTBN2* expression levels above the median level were defined as the high-expression group, whereas those opposite samples were defined as the low-expression group. We applied the Kaplan–Meier method to the data obtained from the TCGA database to analyze the relationship between *SPTBN2* expression levels and the prognosis of patients with LUAD.

#### 2.2.6 Targeted miRNA Prediction

We use several online databases to examine miRNA target prediction and functional annotations. Using the overlapping results obtained from the miRDB, TargetScan, and TarBase databases, assessed using a Venn diagram; we identified predicted miRNAs that target *SPTBN2*. The online prediction website TargetScan was then used to verify the targeted miRNA and identify the *SPTBN2*-binding site targeted by the miRNA sequence. The TargetScan database was also used to analyze the correlation between *SPTBN2* expression level and miRNA expression levels.

### 2.3 Experimental Verification

#### 2.3.1 Cell Culture and Transfection

We purchased human lung cancer cell lines A549 and H1299 from the Shanghai Cell Bank of the Chinese Academy of Sciences. The cells were cultured in Dulbecco’s modified Eagle’s medium (DMEM) in an atmosphere of 5% carbon dioxide at 37°C, supplemented with 10% fetal bovine serum (Invitrogen, USA). The culture medium was changed every 24 h, and cells were passaged every 2–3 days. Small interfering RNA (siRNA) sequences against *SPTBN2* (si-SPTBN2) and negative control (si-SPTBN2 NC) were obtained from Shanghai Jima Pharmaceutical Technology Co., Ltd. (Shanghai, China). The liposome was used for the transient transfection of cell lines, and the transfection process was performed according to the instructions provided with the Lipofectamine 3000 Kit (Invitrogen Company, USA). After 48 h, the cells were collected for real-time fluorescence quantitative polymerase chain reaction (RT-qPCR) to evaluate transfection efficiency, and an siRNA was selected that was able to effectively inhibit the expression of *SPTBN2*.

#### 2.3.2 Real-Time Fluorescence Quantitative Polymerase Chain Reaction

Total RNA was extracted from LUAD tissues, corresponding adjacent tissues, si-SPTBN2–transfected cells, and si-SPTBN2 NC–transfected cells using a total RNA extraction kit, according to the manufacturer’s instructions (TRIzol). The absorbance was measured at 260 and 280 nm to determine the RNA concentration. Reverse transcription was conducted to obtain cDNA according to the instructions included with the reverse transcription kit. The primer sequence for *SPTBN2* was designed and synthesized by Shangya Biotechnology Co., Ltd. (Zhejiang, China). The primer pairs used were 5′-GGAGCGAGATCCCTCCAAAAT-3′ and 5′-GGCTGTTGTCATACTTCTCATGG-3′ for glyceraldehyde 3-phosphate dehydrogenase (*GAPDH*) and 5′-AGTGGCAGAAGCACCAGGCATT-3′ and 5′-TTCTCCGACACCAGGGCTTTCA-3′ for *SPTBN2*. The PCR reaction system was prepared according to the instructions included in the SYBR Premix Ex Taq (TaKaRa Company, Japan) kit. Real-time amplifications were performed in triplicate. The cycling conditions consisted of 1 denaturing cycle at 95°C for 5 min, followed by 40 three-step amplification cycles (incubation at 95°C for 10 s, annealing at 60°C for 30 s, and product elongation and signal acquisition at 72°C for 30 s). *GAPDH* was used as the internal control to calculate the relative expression level, and each experiment was repeated three times.

#### 2.3.3 Immunohistochemistry

Immunohistochemistry (IHC) staining was performed in paraffin-embedded continuous tissue sections. Tissue sections were deparaffinized and rehydrated with xylene and an alcohol gradient. Antigen retrieval was performed using sodium citrate at 121°C for 1 min. Endogenous peroxidase was blocked by immersing the sections in 3% H_2_O_2_ for 20 min, followed by washing with phosphate-buffered saline (PBS, pH 7.4). Sections were blocked with serum at room temperature for 30 min, and then the blocking solution was removed, and the tissues were incubated with primary antibodies against *SPTBN2* (1:500, Santa Cruz) overnight at 4°C. After incubation with the secondary antibody at room temperature for 1 h, immunostaining was visualized with 3,3′-diaminobenzidine (DAB), and nuclei were counterstained with hematoxylin. After sections were dehydrated using an ethanol gradient, the entire stained sections were scanned and analyzed in a panoramic view. Nuclei stained with hematoxylin appeared blue, whereas the DAB reagent developed a brown-yellow color. *SPTBN2* expression was quantified using a histochemistry score (H-score) to examine differences between LUAD tissues and normal tissues [H-Score =∑ (percentage of staining intensity cells × staining intensity)]. Staining intensity was scored as follows: 0 = no color; 1 = faint yellow; 2 = light brown; and 3 = dark brown.

#### 2.3.4 Cell Counting Kit-8 Assay

Cellular proliferation was determined by CCK-8 following the manufacturer’s instructions (Beyotime). Cell suspensions (100 µl, 4,000 cells) of si-SPTBN2 and si-SPTBN2 NC cells were added to each well of a 96-well plate and incubated for 24 h, followed by the addition of 10 µl of the kit reagent (10%), which was incubated in the dark for 1 h. The optical density (OD) was obtained at 450 nm. The CCK-8 kit was used to detect the proliferation of LUAD cells 24, 48, 72, and 96 h after siRNA transfection.

#### 2.3.5 Wound-Healing Assay

Cells were seeded into six-well plates (5 × 10^5^ cells per well) and cultured for 48 h with 10% fetal bovine serum in a 5% carbon dioxide incubator. When the cell confluence was nearly 100%, a scratch was made perpendicular to the cell plane using a sterile pipette tip. The cells were washed three times with sterile PBS to remove non-adherent cells, and the medium was replaced with serum-free medium. The cell migration distance was observed at 0 and 48 h under a microscope.

#### 2.3.6 Transwell Migration and Invasion Assay

The upper chamber of a Transwell chamber was seeded with tumor cells and serum-free medium, and the lower chamber was filled with normal medium containing 10% fetal bovine serum. The rate at which tumor cells migrated to the lower chamber was measured. The invasion experiment required the addition of a Matrigel layer to the upper chamber to simulate the extracellular matrix. Each group of the experimental group and the control group needed three repeated holes, and the number of cells in each hole was 3 × 10^4^. After culturing for 24 h in a 5% carbon dioxide incubator, the cells were fixed with 4% paraformaldehyde for 30 min and stained with 0.1% crystal violet for 30 min. After rinsing with PBS three times, the upper chamber membranes were gently wiped with a cotton swab. The cells were counted under a microscope.

### 2.4 Statistical Analysis

The *SPTBN2* expression in LUAD tissues and normal lung tissues from the GEO database were compared by the Mann–Whitney U test. The Kaplan–Meier method was used to analyze the relationship between the overall survival rate and the *SPTBN2* expression level. GraphPad Prism (version 8) was used to analyze the experimental results of RT-qPCR and CCK-8 analyses, and ImageJ software (version 1.8.0) was used to analyze the wound healing, Transwell migration, and invasion assays. Significance was set as *p <* 0.05.

## 3 Results

### 3.1 The mRNA Expression Levels of *SPTBN2* in Normal Lung and Tumor Tissues

**Figure 1:**
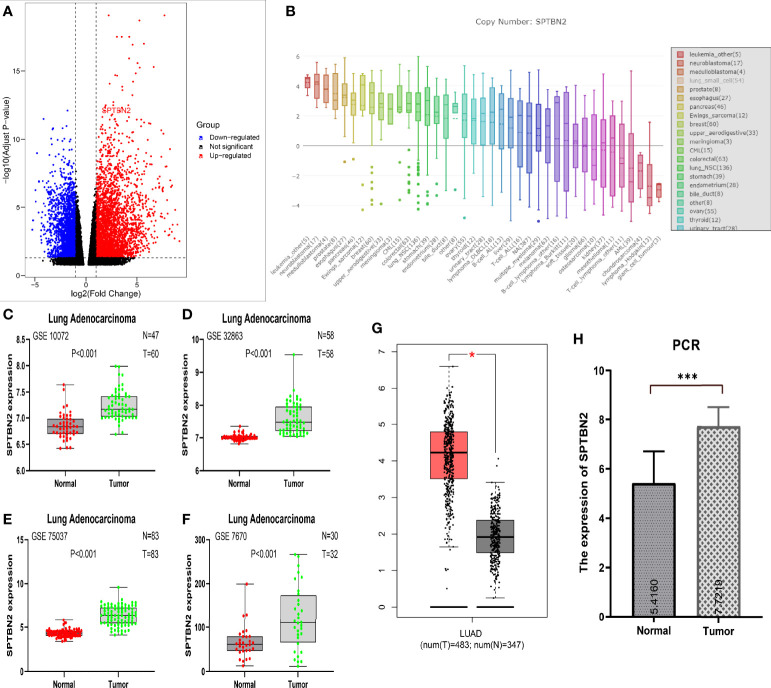
**(A)** RNA sequencing showed the upregulation of β-III-spectrin (*SPTBN2*) in LUAD samples. **(B)** The mRNA expression level of *SPTBN2* in NSCLC ranked 13th among examined cancer cell lines. **(C–H)** The mRNA expression level of *SPTBN2* in LUAD was significantly higher than that in normal tissues, based on GEO, TCGA, and PCR data. *p < 0.05; ***p < 0.001.

The results of RNA-seq revealed the upregulation of *SPTBN2* expression in LUAD samples relative to normal lung tissues (**Figure 1A**). The mRNA expression levels of *SPTBN2* in NSCLC ranked 13th among all examined cancer cell lines (**Figure 1B**). The *SPTBN2* mRNA expression levels in LUAD tissues were significantly higher than those in normal lung tissues from GSE10072 (t = 7.552, p < 0.001), GSE32863 (t = 9.196, p < 0.001), GSE75037 (t = 15.660, p < 0.001), and GSE7670 (t = 3.687, p < 0.001) (**Figures 1C–F**). This upregulation in *SPTBN2* expression was verified using the TCGA database (**Figure 1G**). The relative expression level of *SPTBN2* in 20 LUAD samples was 7.72 ± 0.78, which was significantly higher than that in the adjacent normal tissues (5.42 ± 1.29, t = 6.832, p < 0.001, **Figure 1H**).

### 3.2 PPI Network Analysis and Functional Enrichment Analysis

**Figure 2:**
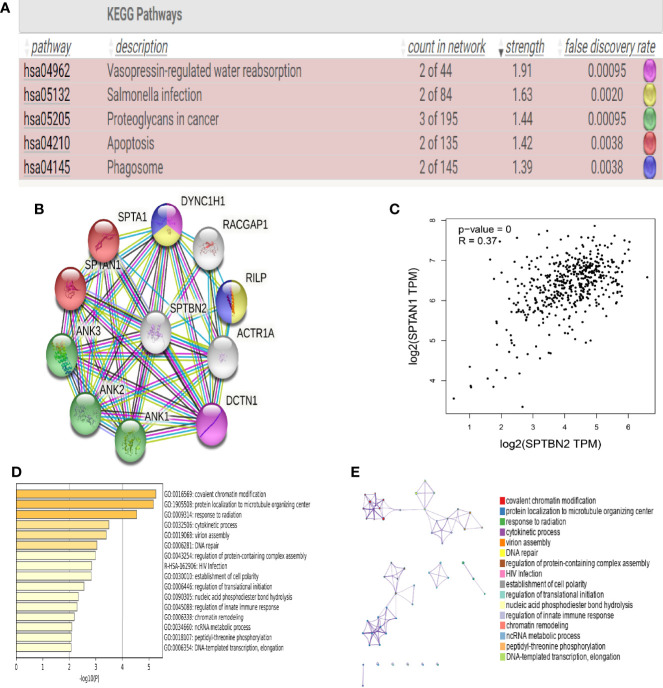
**(A)** Kyoto Encyclopedia of Genes and Genomes (KEGG) pathway analysis indicated that β-III-spectrin (SPTBN2)-related proteins were enriched in apoptotic and phagosomal pathways. **(B, C)** Protein–protein interaction (PPI) network analysis shows that *SPTBN2* has a strong correlation with spectrin alpha, non-erythrocytic 1 (*SPTAN1*). **(D, E)** Histogram and network diagram of gene set enrichment analysis, based on the TCGA database.

Proteins related to *SPTBN2* are significantly enriched in the apoptotic and phagosomal pathways (**Figure 2A**). To evaluate the interactions between differentially expressed genes, a PPI network was constructed based on the STRING database (**Figure 2B**). In the analysis of apoptotic pathways, a strong correlation was identified between *SPTBN2* and spectrin alpha, non-erythrocytic 1 (*SPTAN1*, **Figure 2C**). The top 100 genes with the strongest correlation with *SPTBN2* were enriched and analyzed in the PPI network based on the TCGA database (**Figures 2D, E**).

### 3.3 *SPTBN2* Expression at the Protein Level and Its Impact on Survival

**Figure 3:**
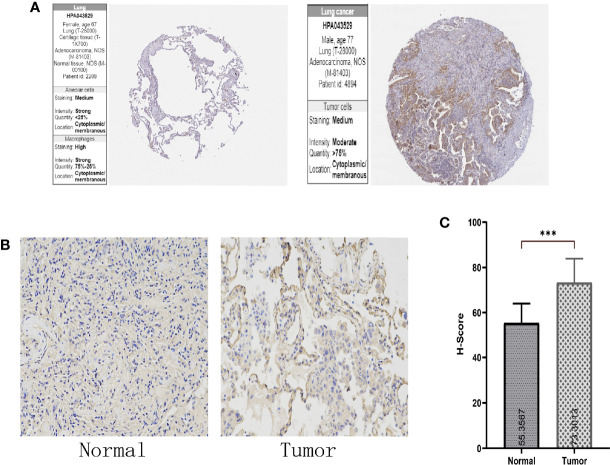
**(A, B)** The Human Protein Atlas (HPA) and immunohistochemistry (IHC) analysis showed that the β-III-spectrin (SPTBN2) protein expression in LUAD tissues was higher than that in normal lung tissues. **(C)** The histochemistry score (H-score) for LUAD tissues was higher than that for normal tissues (***p < 0.001).

**Figure 4:**
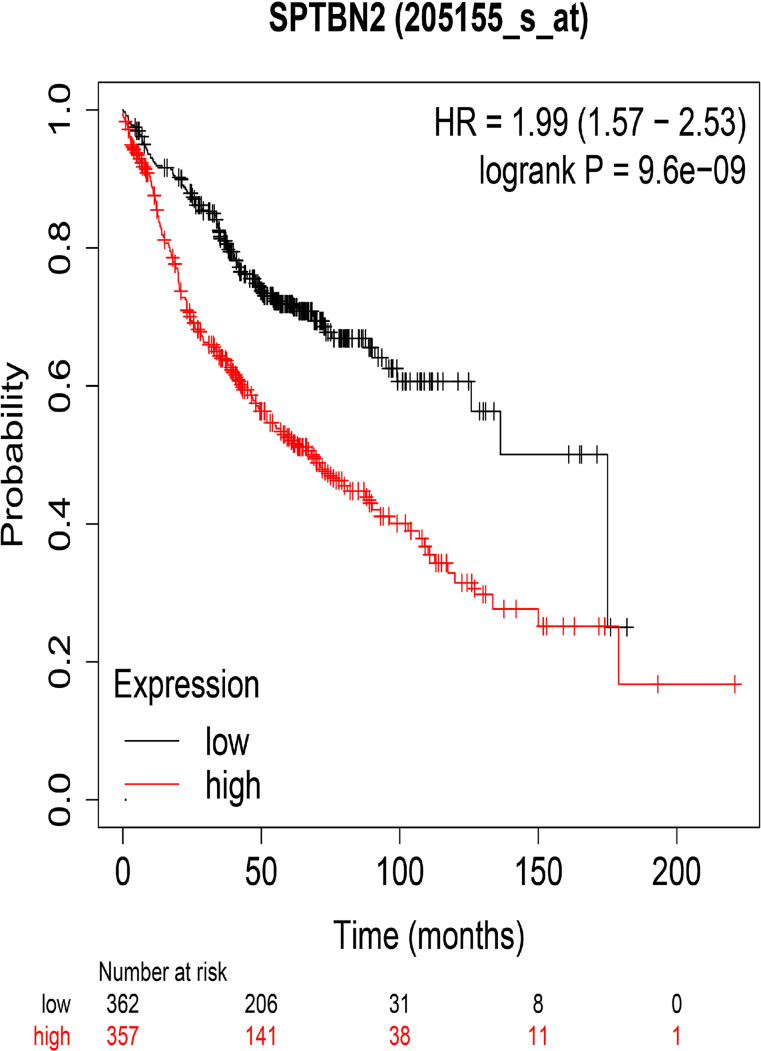
The overall survival rates for groups with high and low levels of β-III-spectrin (*SPTBN2*) expression in LUAD, based on the TCGA database. A high expression of *SPTBN2* was negatively correlated with overall survival in LUAD patients.

The HPA and IHC analyses showed that the *SPTBN2* protein is significantly differentially expressed between tumor and normal tissues (**Figures 3A, B**). The H-score for LUAD tissues was higher than that for normal tissues (p < 0.01, **Figure 3C**). The Kaplan–Meier survival analysis showed that the *SPTBN2* high-expression group was significantly associated with poor prognosis among patients with LUAD (p < 0.001, **Figure 4**).

### 3.4 miR-16 Negatively Regulates the Expression of *SPTBN2* mRNA

**Figure 5:**
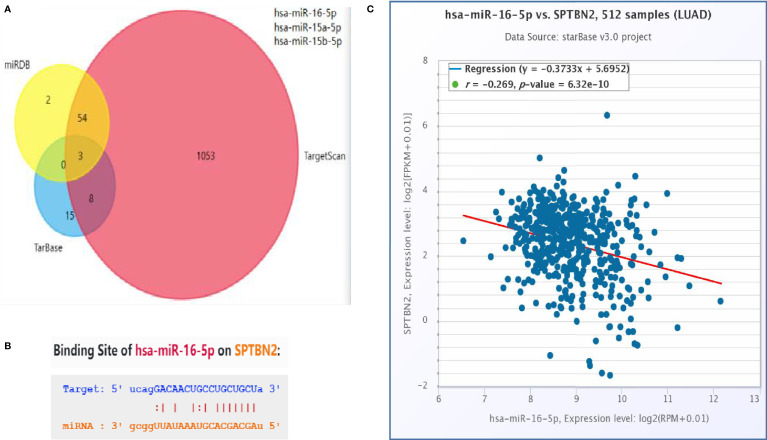
**(A)** The intersection of the outcomes from the miRDB, TargetScan, and TarBase databases. **(B)** Possible binding sites between hsa-miR-16-5p and β-III-spectrin (*SPTBN2*). **(C)** Correlation between *SPTBN2* expression and hsa-miR-16-5p expression in lung adenocarcinoma tissues.

A Venn diagram was used to identify overlapping *SPTBN2*-targeting miRNAs identified by the miRDB, TargetScan, and TarBase databases (**Figure 5A**). TargetScan was used to verify miR-16 as an *SPTBN2*-targeting miRNA, and the binding site for *SPTBN2* was found in the sequence of miR-16 (**Figure 5B**). In addition, the correlation between the *SPTBN2* expression level and the miR-16 expression was analyzed using the TargetScan database. The expression level of *SPTBN2* was significantly negatively correlated with the expression level of miR-16 (**Figure 5C**).

### 3.5 Cell Proliferation, Migration, and Invasion Assays

**Figure 6:**
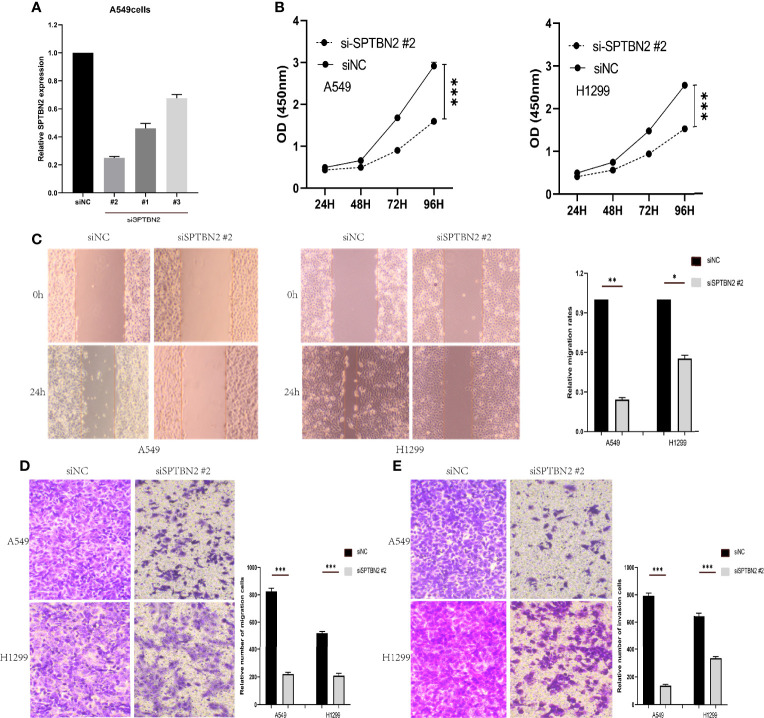
**(A)** Small interfering RNAs (siRNAs) were assessed for the ability to effectively inhibit β-III-spectrin (*SPTBN2*) expression, and the knockdown efficiency was evaluated by real-time fluorescence quantitative polymerase chain reaction (RT-qPCR). **(B)** The cell counting kit-8 (CCK-8) proliferation assay demonstrated a significant decrease in the proliferation of the Si-SPTBN2 group compared with the Si-SPTBN2-negative control (NC) group. **(C)** The scratch experiment showed that *SPTBN2* knockdown resulted in a shorter vertical migration distance compared with the control group after 48 h. **(D, E)** Transwell assay showed that the migration and invasion abilities of the Si-SPTBN2 group decreased compared with those of the Si-SPTBN2 NC group. *p < 0.05; **p < 0.01; ***p < 0.001.

The siRNA molecules were screened to identify those able to effectively inhibit the expression of *SPTBN2* (**Figure 6A**). As shown in **Figure 6B**, the CCK-8 proliferation assay performed in A549 cells showed that si-SPTBN2 transfection significantly decreased cell proliferation (**Table 1**). CCK-8 proliferation assay showed similar results in H1299 cells, with *SPTBN2* knockdown associated with significantly decreased cell proliferation ability (**Table 2**). The scratch experiment showed that the knockdown of *SPTBN2* reduced the vertical migration distance compared with the control group 48 h after scratching (**Figure 6C**). The Transwell assay revealed that migration (A549 cells: 221 ± 12 cells *vs*. 825 ± 23 cells, t = 45.470, p < 0.001; H1299 cells: 210 ± 16 cells *vs*. 520 ± 12 cells, t = 26.850, p < 0.001) and invasion (A549 cells: 137 ± 10 cells *vs*. 792 ± 20 cells, t = 50.740, p < 0.001; H1299 cells: 340 ± 11 cells *vs*. 647 ± 21 cells, t = 22.430, p < 0.001) were significantly decreased in LUAD cells following *SPTBN2* knockdown compared with the control group (**Figures 6D, E**).

**Table 1:** Proliferation ability of A549 cells.

Time	si-SPTBN2 group (OD)	Control group (OD)	t-value	p-value
24 h	0.44 ± 0.02	0.50 ± 0.01	6.050	p < 0.01
48 h	0.49 ± 0.01	0.66 ± 0.02	11.022	p < 0.001
72 h	0.90 ± 0.03	1.68 ± 0.01	40.730	p < 0.001
96 h	0.91 ± 0.02	2.91 ± 0.09	30.674	p < 0.001

SPTBN2, β-III-spectrin.

**Table 2:** Proliferation ability of H1299 cells.

Time	si-SPTBN2 group (OD)	Control group (OD)	t-value	p-value
**24 h**	0.42 ± 0.02	0.49 ± 0.01	6.003	p < 0.01
**48 h**	0.50 ± 0.01	0.68 ± 0.03	10.719	p < 0.001
**72 h**	0.91 ± 0.03	2.11 ± 0.01	68.658	p < 0.001
**96 h**	0.93 ± 0.05	3.00 ± 0.01	51.363	p < 0.001

SPTBN2, β-III-spectrin.

## 4 Discussion

Currently, lung cancer is among the most common cancers threatening human health and remains the most common cause of cancer-related death (16). Morbidity and mortality associated with lung cancer continue to increase yearly (17). The 5-year relative survival rate of patients diagnosed with lung cancer is 19% (18). According to the histological findings, lung cancer can be divided into two main subtypes, small cell lung cancer (SCLC), and NSCLC, which account for 15% and 85%, respectively, of all lung cancer cases (19). The most common histological lung cancer subtype is LUAD (20), which accounts for 50% of all NSCLC cases (21). LUAD develops from small airway epithelial type II alveolar cells, which secrete mucus and other substances (22), and LUAD cases are the primary causes of human deaths due to lung cancer. Compared with other lung cancer subtypes, LUAD grows more slowly, with no distinct clinical manifestations during early stages. Approximately 75% of patients have entered the advanced stage by the time of diagnosis, at which point radical treatment is no longer an option (23). Patients with early-stage IA cancer have a 75% chance of surviving for longer than 5 years (24). Therefore, improved lung cancer detection at an early stage could dramatically reduce lung cancer mortality. Although the survival rate of LUAD has improved with advancements in medical standards and the emergence of new drugs and immunotherapies (25), the overall survival rate and quality of life among patients remain unsatisfactory. In recent years, in addition to improving diagnosis and treatment based on existing knowledge, the identification of effective early-stage tumor markers has become a focus of LUAD research.

*SPTBN2*, located on chromosome 11, encodes spectrin beta, non-erythrocytic 2, also known as beta-III spectrin (26). Spectrins are composed of two alpha and two beta spectrin subunits and are vital components of a cell’s membrane–cytoskeleton connection, providing shape, strength, and elasticity by connecting cytoskeleton components with the plasma membrane through protein–protein and protein–lipid interactions (27–29). *SPTBN2* regulates the glutamate signaling pathway by stabilizing excitatory amino-acid transporter 4 (EAAT4), a glutamate transporter expressed on the surface of the plasma membrane (3). Mutations in *SPTBN2* can lead to a form of spinocerebellar ataxia (SCA5), which is characterized by neurodegeneration, progressive locomotor incoordination, dysarthria, and uncoordinated eye movements (3, 30, 31). Beta-III spectrin is a widely secreted plasma protein that can be detected in a variety of tissues and cells. In recent years, increasing evidence has indicated that *SPTBN2* is highly expressed in various tumors and plays considerable roles in tumor occurrence and metastasis. Yang et al. (32) indicated that *SPTBN2* was a potential driver gene in breast cancer and endometrial cancer. Huang et al. (33) identified the significant upregulation of *SPTBN2* expression in bladder cancer tumor samples compared with normal samples, and *SPTBN2* expression was associated with a poor prognosis in bladder cancer. Zhang et al. (34) discovered that the expression of *SPTBN2* was significantly upregulated in colorectal cancer patients with distant metastasis. In addition, Ma et al. (35) reported that high *SPTBN2* expression was associated with a worse prognosis in colorectal cancer patients. However, the relationship between *SPTBN2* and LUAD has not been reported.

In this study, we found that the expression of *SPTBN2* was increased in LUAD compared with normal lung tissues. High *SPTBN2* expression has an adverse effect on the overall survival rate of LUAD patients. Our data revealed that silencing *SPTBN2* inhibited cell proliferation, migration, and invasion abilities. The purpose of this study was to explore the correlation between *SPTBN2* expression and LUAD prognosis and to determine whether *SPTBN2* might serve as a target for novel diagnostic or therapeutic approaches in LUAD. Based on our findings, we conclude that *SPTBN2* can serve as a biological marker of LUAD, acts as an oncogenic gene, and is a potential therapeutic target for LUAD therapy.

## Data Availability Statement

Publicly available datasets were analyzed in this study, which can be found here: https://www.frontiersin.org/articles/10.3389/fcell.2021.739358/full#.

## Ethics Statement

The studies involving human participants were reviewed and approved by the Ethics Committee of the First Affiliated Hospital of Zhengzhou University (No. 2019-KY-255). The patients/participants provided their written informed consent to participate in this study.

## Author Contributions

CW and BD designed and conducted the experiments and wrote the manuscript. YL conducted the Western blot analysis. GY conducted the cell proliferation assay and flow cytometry. SL conducted the quantitative PCR. LH and YQ conceptualized and supervised all aspects of the studies. All authors contributed to the article and approved the submitted version.

## Conflict of Interest

The authors declare that the research was conducted in the absence of any commercial or financial relationships that could be construed as a potential conflict of interest.

## Publisher’s Note

All claims expressed in this article are solely those of the authors and do not necessarily represent those of their affiliated organizations, or those of the publisher, the editors and the reviewers. Any product that may be evaluated in this article, or claim that may be made by its manufacturer, is not guaranteed or endorsed by the publisher.

## References

[1] Bray F Ferlay J Soerjomataram I Siegel RL Torre LA Jemal A. Global Cancer Statistics 2018: GLOBOCAN Estimates of Incidence and Mortality Worldwide for 36 Cancers in 185 Countries. CA Cancer J Clin (2018) 68(6):394–424. doi: 10.3322/caac.21492 30207593

[2] Jackson M Song W Liu MY Jin L Dykes-Hoberg M Lin CI. Modulation of the Neuronal Glutamate Transporter EAAT4 by Two Interacting Proteins. Nature (2001) 410(6824):89–93. doi: 10.1038/35065091 11242047

[3] Dick KA Ikeda Y Day JW Ranum LP. Spinocerebellar Ataxia Type 5. Handb Clin Neurol (2012) 103:451–9. doi: 10.1016/B978-0-444-51892-7.00028-0 21827906

[4] Chen J Yao ZX Chen JS Gi YJ Muñoz NM Kundra S. TGF-β/β2-Spectrin/CTCF-Regulated Tumor Suppression in Human Stem Cell Disorder Beckwith-Wiedemann Syndrome. J Clin Invest (2016) 126(2):527–42. doi: 10.1172/JCI80937 PMC4731168 26784546

[5] Barretina J Caponigro G Stransky N Venkatesan K Margolin AA Kim S. The Cancer Cell Line Encyclopedia Enables Predictive Modelling of Anticancer Drug Sensitivity. Nature (2012) 483(7391):603–7. doi: 10.1038/nature11003 PMC3320027 22460905

[6] Clough E Barrett T. The Gene Expression Omnibus Database. Methods Mol Biol (2016) 1418:93–110. doi: 10.1007/978-1-4939-3578-9_5 27008011 PMC4944384

[7] Teresa LM Tatiana D Melissa R Figueroa JD Liu H Abhijit D. Gene Expression Signature of Cigarette Smoking and Its Role in Lung Adenocarcinoma Development and Survival. PLoS One (2008) 3(2):e1651. doi: 10.1371/journal.pone.0001651 18297132 PMC2249927

[8] Selamat SA Chung BS Girard L Zhang W Zhang Y Campan M. Genome-Scale Analysis of DNA Methylation in Lung Adenocarcinoma and Integration With mRNA Expression. Genome Res (2012) 22(7):1197–211. doi: 10.1101/gr.132662.111 PMC3396362 22613842

[9] Girard L Rodriguez-Canales J Behrens C Thompson DM Botros IW Tang H. An Expression Signature as an Aid to the Histologic Classification of Non-Small Cell Lung Cancer. Clin Cancer Res (2016) 22(19):4880–9. doi: 10.1158/1078-0432.CCR-15-2900 PMC5492382 27354471

[10] Su LJ Chang CW Wu YC Chen KC Lin CJ Liang SC. Selection of DDX5 as a Novel Internal Control for Q-RT-PCR From Microarray Data Using a Block Bootstrap Re-Sampling Scheme. BMC Genomics (2007) 8:140. doi: 10.1186/1471-2164-8-140 17540040 PMC1894975

[11] Tang Z Li C Kang B Gao G Li C Zhang Z. GEPIA: A Web Server for Cancer and Normal Gene Expression Profiling and Interactive Analyses. Nucleic Acids Res (2017) 45(W1):W98–102. doi: 10.1093/nar/gkx247 28407145 PMC5570223

[12] Uhlén M Björling E Agaton C Szigyarto CA Amini B Andersen E. A Human Protein Atlas for Normal and Cancer Tissues Based on Antibody Proteomics. Mol Cell Proteomics (2005) 4(12):1920–32. doi: 10.1074/mcp.M500279-MCP200 16127175

[13] Uhlen M Oksvold P Fagerberg L Lundberg E Jonasson K Forsberg M. Towards a Knowledge-Based Human Protein Atlas. Nat Biotechnol (2010) 28(12):1248–50. doi: 10.1038/nbt1210-1248 21139605

[14] Uhlén M Fagerberg L Hallström BM Lindskog C Oksvold P Mardinoglu A. Proteomics. Tissue-Based Map of the Human Proteome. Science (2015) 347(6220):1260419. doi: 10.1126/science.1260419 25613900

[15] Silva TC Colaprico A Olsen C D’Angelo F Bontempi G Ceccarelli M. TCGA Workflow: Analyze Cancer Genomics and Epigenomics Data Using Bioconductor Packages. F1000Res (2016) 5:1542. doi: 10.12688/f1000research.8923.1 28232861 PMC5302158

[16] Cai Z Liu Q. Understanding the Global Cancer Statistics 2018: Implications for Cancer Control. Sci China Life Sci (2021) 64(6):1017–20. doi: 10.1007/s11427-019-9816-1 31463738

[17] Barta JA Powell CA Wisnivesky JP. Global Epidemiology of Lung Cancer. Ann Glob Health (2019) 85(1):8. doi: 10.5334/aogh.2419 30741509 PMC6724220

[18] Siegel RL Miller KD Jemal A. Cancer Statistics, 2019. CA Cancer J Clin (2019) 69(1):7–34. doi: 10.3322/caac.21551 30620402

[19] Sher T Dy GK Adjei AA. Small Cell Lung Cancer. Mayo Clin Proc (2008) 83(3):355–67. doi: 10.4065/83.3.355 18316005

[20] Travis WD Brambilla E Noguchi M Nicholson AG Geisinger KR Yatabe Y. International Association for the Study of Lung Cancer/American Thoracic Society/European Respiratory Society International Multidisciplinary Classification of Lung Adenocarcinoma. J Thorac Oncol (2011) 6(2):244–85. doi: 10.1097/JTO.0b013e318206a221 PMC4513953 21252716

[21] Peng Z Wang J Shan B Li B Peng W Dong Y. The Long Noncoding RNA LINC00312 Induces Lung Adenocarcinoma Migration and Vasculogenic Mimicry Through Directly Binding Ybx1. Mol Cancer (2018) 17(1):167. doi: 10.1186/s12943-018-0920-z 30470227 PMC6260658

[22] Noguchi M Morikawa A Kawasaki M Matsuno Y Yamada T Hirohashi S. Small Adenocarcinoma of the Lung. Histol Characteristics Prognosis Cancer (1995) 75(12):2844–52. doi: 10.1002/1097-0142(19950615)75:123.0.CO 7773933

[23] Zappa C Mousa SA. Non-Small Cell Lung Cancer: Current Treatment and Future Advances. Transl Lung Cancer Res (2016) 5(3):288–300. doi: 10.21037/tlcr.2016.06.07 27413711 PMC4931124

[24] Goldstraw P Chansky K Crowley J Rami-Porta R Asamura H Eberhardt WE. The IASLC Lung Cancer Staging Project: Proposals for Revision of the TNM Stage Groupings in the Forthcoming (Eighth) Edition of the TNM Classification for Lung Cancer. J Thorac Oncol (2016) 11(1):39–51. doi: 10.1016/j.jtho.2015.09.009 26762738

[25] Camidge DR Doebele RC Kerr KM. Comparing and Contrasting Predictive Biomarkers for Immunotherapy and Targeted Therapy of NSCLC. Nat Rev Clin Oncol (2019) 16(6):341–55. doi: 10.1038/s41571-019-0173-9 30718843

[26] Wen JX Li XQ Chang Y. Signature Gene Identification of Cancer Occurrence and Pattern Recognition. J Comput Biol (2018) 25(8):907–16. doi: 10.1089/cmb.2017.0261 29957033

[27] Zhang R Zhang C Zhao Q Li D. Spectrin: Structure, Function and Disease. Sci China Life Sci (2013) 56(12):1076–85. doi: 10.1007/s11427-013-4575-0 24302288

[28] Zhang C Rasband MN. Cytoskeletal Control of Axon Domain Assembly and Function. Curr Opin Neurobiol (2016) 39:116–21. doi: 10.1016/j.conb.2016.05.001 PMC4987246 27203619

[29] Machnicka B Czogalla A Hryniewicz-Jankowska A Bogusławska DM Grochowalska R Heger E. Spectrins: A Structural Platform for Stabilization and Activation of Membrane Channels, Receptors and Transporters. Biochim Biophys Acta (2014) 1838(2):620–34. doi: 10.1016/j.bbamem.2013.05.002 23673272

[30] Avery AW Crain J Thomas DD Hays TS. A Human β-III-Spectrin Spinocerebellar Ataxia Type 5 Mutation Causes High-Affinity F-Actin Binding. Sci Rep (2016) 6:21375. doi: 10.1038/srep21375 26883385 PMC4756369

[31] Romaniello R Citterio A Panzeri E Arrigoni F De Rinaldis M Trabacca A. Novel SPTBN2 Gene Mutation and First Intragenic Deletion in Early Onset Spinocerebellar Ataxia Type 5. Ann Clin Transl Neurol (2021) 8(4):956–63. doi: 10.1002/acn3.51345 PMC8045899 33756041

[32] Yang Z Yu G Guo M Yu J Zhang X Wang J. CDPath: Cooperative Driver Pathways Discovery Using Integer Linear Programming and Markov Clustering. IEEE/ACM Trans Comput Biol Bioinform (2021) 18(4):1384–1395. doi: 10.1109/TCBB.2019.2945029 31581094

[33] Huang M Long Y Jin Y Ya W Meng D Qin T. Comprehensive Analysis of the lncRNA-miRNA-mRNA Regulatory Network for Bladder Cancer. Transl Androl Urol (2021) 10(3):1286–301. doi: 10.21037/tau-21-81 PMC8039630 33850763

[34] Zhang Z Wang Q Zhang M Zhang W Zhao L Yang C. Comprehensive Analysis of the Transcriptome-Wide M6a Methylome in Colorectal Cancer by MeRIP Sequencing. Epigenetics (2021) 16(4):425–35. doi: 10.1080/15592294.2020.1805684 PMC7993153 32749190

[35] Ma J Wang P Huang L Qiao J Li J. Bioinformatic Analysis Reveals an Exosomal miRNA-mRNA Network in Colorectal Cancer. BMC Med Genomics (2021) 14(1):60. doi: 10.1186/s12920-021-00905-2 33639954 PMC7913431

